# Development of Adenosine Deaminase-Specific IgY Antibodies: Diagnostic and Inhibitory Application

**DOI:** 10.1007/s12010-017-2626-x

**Published:** 2017-10-17

**Authors:** Agnieszka Łupicka-Słowik, Mateusz Psurski, Renata Grzywa, Kamila Bobrek, Patrycja Smok, Maciej Walczak, Andrzej Gaweł, Tadeusz Stefaniak, Józef Oleksyszyn, Marcin Sieńczyk

**Affiliations:** 1 Faculty of Chemistry, Division of Medicinal Chemistry and Microbiology, Wroclaw University of Science and Technology, Wybrzeże Wyspiańskiego 27, 50-370 Wrocław, Poland; 2 Laboratory of Experimental Anticancer Therapy, Department of Experimental Oncology, Ludwik Hirszfeld Institute of Immunology and Experimental Therapy, Polish Academy of Sciences, Weigla 12, 53-114 Wrocław, Poland; 3 Faculty of Veterinary Medicine, Department of Epizootiology and Clinic of Bird and Exotic Animals, Wroclaw University of Environmental and Life Sciences, Pl. Grunwaldzki 45, 50-366 Wrocław, Poland; 4 Faculty of Veterinary Medicine, Department of Immunology, Pathophysiology and Veterinary Preventive Medicine, Wroclaw University of Environmental and Life Sciences, Norwida 31, 50-375 Wrocław, Poland

**Keywords:** Egg yolk immunoglobulin (IgY), Adenosine deaminase (ADA), Anti-adenosine deaminase antibody, Affinity purification, Enzyme inhibition, ELISA

## Abstract

Adenosine deaminase (ADA) is currently used as a diagnostic marker for tuberculous pleuritis. Although ADA has been suggested as a potential marker for several types of cancer, the importance of each of ADA isoforms as well as their levels and enzymatic activities in tumors need to be further investigated. Herein we developed avian immunoglobulin Y highly specific to human ADA via hens immunization with calf adenosine deaminase. The obtained antibodies were used for the development of a sensitive double-egg yolk immunoglobulin (IgY) sandwich ELISA assay with an ADA detection limit of 0.5 ng/ml and a linearity range of up to 10 ng/ml. Specific, affinity-purified IgYs were able to recognize human recombinant ADA and ADA present in human cancer cell lines. In addition, antigen-specific IgY antibodies were able to inhibit catalytic activity of calf ADA with an IC_50_ value of 47.48 nM. We showed that generated IgY antibodies may be useful for ADA detection, thus acting as a diagnostic agent in immunoenzymatic assays.

## Introduction

In human body fluids and tissues, adenosine deaminase (adenosine aminohydrolase, EC 3.5.4.4) is present as two isozymes: ADA_1_ and ADA_2_. Both isozymes play an essential role in purine nucleoside metabolism catalyzing the irreversible deamination reaction of adenosine or 2′deoxyadenosine to inosine or 2′deoxyinosine. These two isozymes are kinetically distinguishable by their reaction with EHNA ((+)-erythro-9-(2-hydroxy-3-nonyl)adenine), a specific inhibitor of ADA_1_ [1].

The ADA_1_ isozyme is ubiquitous in all human tissues and erythrocytes with a small amount circulating in plasma [2]. ADA_1_ exists in two molecular forms: the small monomeric adenosine deaminase (ADA_1_-S, 41 kDa) and the large adenosine deaminase (ADA_1_-L, 298 kDa), which is composed of ADA_1_-S and adenosine-binding protein (dipeptidyl-dipeptidase IV (DPPIV) or CD26) [3–6]. The small globular ADA_1_-S molecule is formed by a characteristic parallel α/β-barrel motif (TIM barrel fold) with a zinc cofactor located in the catalytic pocket [7]. Interacting with ADA, dipeptidyl peptidase mediates co-stimulatory signals in T-lymphocytes. The protein-protein contact between these two molecules occurs through two hydrophobic loops in the β-propeller domain of DPPIV and two hydrophilic α-helices within ADA, which is why even after the formation of the complex both enzymes remain catalytically active [8].

The second isozyme [ADA_2_] belongs to the adenosine deaminase growth factor family. It is found in monocytes and represents the main ADA isozyme present in human plasma and serum (originating from monocytes-macrophages) [9]. ADA_2_ exists as an extensively glycosylated functional homodimer (110 kDa) with a signal peptide and a conserved disulfide bond [4, 10, 11]. According to Zavialov et al., the ADA_1_-like domain of the ADA_2_ isozyme shares approximately 70% of its total amino acid sequence similarity with the ADA_1_ protein, but with only 20% of amino acid identity [11].

According to experimental data presented by Kelly et al., there is 93% sequence identity between bovine and human ADA [12]. Simple sequence alignment using the protein Basic Local Alignment Search Tool (BLAST) algorithm indicates no gaps between the two sequences, and 91% of amino acid residues are identical (94% represent positives: 1VFL.pdb and 3IAR.pdb). The high homology of these proteins was the reason we selected calf ADA as a target protein for the development of egg yolk immunoglobulin (IgY) antibodies described in this manuscript.

In a healthy organism, the tissue and blood concentration of adenosine, an important signaling metabolite, is low and its extracellular physiological level does not exceed 1 μM [13, 14]. Local adenosine concentration increases significantly during inflammation, ischemia, or hypoxia and can reach 100 μM [14–16]. Adenosine’s effect is contingent on the cell type: it may serve as a cytoprotective agent, stimulate angiogenesis, and decrease inflammation. On the other hand, the necrosis- and hypoxia-induced release of adenosine may result in the enhancement of angiogenesis and promotion of tumor growth [17]. An increased activity of ADA in malignant tissues is associated with their compensatory mechanism against toxic levels of adenosine, deoxyadenosine, and its derivatives which are potent inhibitors of ribonucleotide reductase (RNR), a rate-limiting step of nucleotide biosynthesis [18]. The activity of RNR is particularly important for cells that undergo division [19]. ADA-catalyzed deamination of adenosine and 2′deoxyadenosine leads decreased levels of intermediates acting as RNR inhibitors. Therefore, some ADA inhibitors (such as erythro-9-(2-hydroxy-3-nonyl)adenine, EHNA) induce apoptosis of malignant tumor cell lines and suppress tumor growth by increasing intracellular adenosine/deoxyadenosine concentration [20]. Furthermore, pentostatin (2′-deoxycoformycin), approved by the FDA for the treatment of chronic B-cell lymphoproliferative disorders, is a nucleoside analog which non-competitively inhibits ADA and leads to an accumulation of adenosine metabolites that inhibit RNR [21].

An increased serum level/activity of ADA is often observed during the development of breast, bladder, ovarian, head and neck, and laryngeal cancer [22–27]. An increased activity of ADA was also observed in cancerous colorectal tissues as well as in saliva from patients suffering tongue squamous cell carcinoma [28, 29]. ADA as a diagnostic marker is routinely used for the diagnosis of tuberculous pleuritis with a high degree of specificity and sensitivity [30, 31]. It is important to highlight that different isozymes/molecular forms of ADA have been found in tumor cells, which may indicate that the composition of isoforms during cancer progression may change [32, 33].

Considering the advantages of IgY antibodies as diagnostic tools, we decided to generate ADA_1_-specific avian IgYs. After immunization, the IgY class of immunoglobulins is isolated from chicken egg yolks, so in contrast to the production of mammalian antibodies, no animal bleeding is necessary. Additionally, the cost of IgY antibodies is significantly lower than that of their mammalian equivalents without deteriorating their quality [34, 35]. The IgY technology makes it possible to obtain more than 1000 mg of antibodies (of which 2–10% are antigen-specific) from one hen within 2 weeks while a parallel isolation of rabbit IgG yields approximately 200 mg with 5% antigen-specific immunoglobulins [36]. The use of IgY antibodies in serological diagnostics reduces the risk of false-positive results, since IgYs do not interact with the rheumatoid factor, components of the complement system, or human anti-mouse IgG [37]. Indeed, the incidence of false results may be as high as 12%, thus the reduction of false positives is crucial for improved serum diagnostics [38]. A considerable advantage of antibodies generated in the hen as a host organism is the evolutionary distance between mammals and birds. It is rather challenging to obtain antibodies specific towards conserved mammalian antigens in mammalian systems whereas the same antigens are highly immunogenic for hens [39].

Until now, hen immunoglobulins have been developed as diagnostic tools for infectious agents including *Listeria monocytogenes*, *Escherichia coli*, and *Mycobacterium avium* subsp. *paratuberculosis* as well as tumor markers such as thymidine kinase 1, Human epidermal growth factor family receptor-2/Neu (HER2) and telomerase, kallikrein 6 (KLK6), cancer antigen 15-3 (CA 15-3), and KLK3 [40–47]. IgY antibodies can also be used as neutralizing, anti-toxin agents and for passive immunization. Specific IgY antibodies obtained after immunization with the recombinant Shiga toxin-2 (Stx2) subunit are able to effectively block the biological activity of Stx2, one of the main virulence factors of *E. coli* [41]*.* IgYs specific to *Solobacterium moorei* exhibit the potential to inhibit their growth and biofilm formation [48]. At present, as an alternative to mammalian anti-sera, avian immunoglobulins are produced as an anti-venom agent neutralizing Naja, *Bitis*, coral snake, and Brazilian *Bothrops* toxins [49–52].

Considering the importance of ADA as a disease marker, we have decided to generate avian antibodies and develop an IgY-based sandwich-type ELISA assay for a specific and sensitive detection of ADA. Additionally, anti-calf adenosine deaminase (cADA) IgY antibodies were found to be potent inhibitors of enzymatic activity of ADA.

## Materials and Methods

### Immunization and Antibody Isolation

Immunization of hens and isolation of IgY antibodies were performed as follows: 22-week-old *White Leghorn* egg-laying hens were purchased from a commercial source (Woźniak Poultry Farm, Żylice, Poland) and randomly split into two groups containing four hens each. One group received an antigen with Freund’s complete adjuvant (MP Biomedicals, Solon, OH, USA), while the control group received only an adjuvant solution. The native calf ADA (100 μg; cADA, Roche, Warsaw, Poland) was dissolved in 150 μl of 0.9% saline (Baxter, Warsaw, Poland) and emulsified with an equal amount of Freund’s adjuvant. Animals were immunized intramuscularly (*Musculus pectoralis*, left and right) at two different sites with 150 μl per site. The booster injections were administered after 4 and 8 weeks of primary immunization (100 μg/animal, in Freund’s incomplete adjuvant) [46, 47].

The isolation of IgY antibodies from eggs collected daily was conducted separately for each egg according to the PEG 6000 precipitation method described by Polson et al. with slight modifications [46, 53]. The purity of the obtained IgY antibodies was examined by non-reducing SDS-PAGE (4–12%, Tris-glycine) followed by Coomassie R250 staining (Calbiochem, Warsaw, Poland).

### Antigen-Specific IgY Antibody Production

We used Western blot and ELISA to examine the development of hen immune response over the course of immunization as manifested by the production of specific IgYs and increased antibody avidity. For Western blot assay, calf ADA (50 ng/lane) was resolved by SDS-PAGE (4–12%, Tris-glycine) under reducing conditions and blotted (semi-dry blotting system, Cleaver Scientific, Rugby, UK) onto nitrocellulose membrane (0.45 μm, Thermo Scientific, Gdańsk, Poland). The membrane was blocked with 5% skim milk in 10 mM phosphate-buffered saline with 0.05% Tween-20, pH 7.4 (PBST; 4 °C, overnight). Next, the membrane was washed with PBST (20 min, three times) and cut into strips which were further incubated with IgY antibodies diluted 1:100 in 0.5% skim milk in PBST (1 h, 37 °C). After washing the membrane with PBST, the detection of the resulting antigen-antibody complexes was carried out with anti-IgY rabbit IgG antibodies conjugated with horseradish peroxidase (Pierce, Gdańsk, Poland) diluted 1:5000 in 0.5% skim milk in PBST. Following a 1-h incubation at 37 °C, the membrane was washed and the signal was developed with a chemiluminescent peroxidase substrate (West Pico, Pierce, Gdańsk, Poland) and the bands were visualized with the blot imaging system (Gel Logic 1500, Carestream, Rochester, NY, USA).

For an indirect ELISA, 96-well microtiter plates (MaxiSorp, Nunc, Gdańsk, Poland) were coated with calf ADA (0.5 μg/ml, 100 μl/well) in 50 mM sodium carbonate buffer, pH 9.6, followed by 3 h of incubation at 37 °C. The plates were further washed three times with PBST, and a solution of IgY antibodies isolated from egg yolks prepared in 0.5% skim milk in PBST (1:100, 100 μl/well) was added. After 1 h of incubation at 37 °C, the plates were washed with PBST as before and incubated either with 6 M urea in PBST or with PBST for 10 min at room temperature. For the detection of ADA-IgY antibody complexes, the plates were washed and rabbit anti-IgY IgG-HRP antibodies were added into each well (1:5000, prepared in 0.5% skim milk in PBST, 100 μl/well). After washing the plates, the peroxidase substrate solution (*O*-phenylenediamine, OPD, Pierce, Gdańsk, Poland) in 50 mM citrate buffer, pH 5.0, supplemented with 0.015% H_2_O_2_ was added (100 μl/well). The reaction was quenched by the addition of 1 M H_2_SO_4_ (50 μl/well), and absorbance at 490 nm was measured using the microplate reader (Multiskan FC, Thermo Scientific, Gdańsk, Poland). The results were expressed as the OD_490_^*^ values obtained after the subtraction of the values taken for the control antibodies. All measurements were performed in duplicate.

### Titer of IgY Antibodies

The antigen-specific IgY antibody titers were determined by Western blot and ELISA analysis. For Western blot, calf ADA protein (50 ng/lane) was resolved by SDS-PAGE and blotted as described above. After blocking (4 °C, overnight), the membrane was washed in PBST and cut into strips which were incubated with the cADA-specific IgY antibody solutions prepared in 0.5% skim milk in PBST at concentrations of 50, 10, 5, 1, and 0.1 μg/ml or with control IgY antibodies (50 μg/ml) for 1 h at 37 °C. Next, the membrane strips were washed with PBST and the secondary peroxidase-conjugated antibodies were used as previously described.

For indirect ELISA assay, a microtiter plate (MaxiSorp, Nunc, Gdańsk, Poland) was coated with cADA (0.5 μg/ml, 100 μl/well) in 50 mM sodium carbonate buffer, pH 9.6 (1 h, 37 °C). The plates were washed with PBST and blocked with 5% skim milk in PBST. The wells were then washed with PBST, and the antigen-specific or control IgY antibodies at different concentrations were added (50, 10, 5, 1, 0.1 μg/ml). After 1 h of incubation (37 °C), the plate was washed and secondary antibodies were applied (anti-IgY rabbit IgG-HRP antibodies, 1:5000 in 0.5% skim milk/PBST). The plate was developed with OPD solution as described above. The results were expressed as the ELISA index (EI), where EI = OD_sample_/OD_control_, with values of EI > 1.2 considered as positive [54].

### Calf ADA Detection Limit

Different amounts ranging from 50 to 0.1 ng/lane of ADA were resolved by SDS-PAGE under reducing conditions and blotted onto a nitrocellulose membrane. After blocking (5% skim milk in PBST, 4 °C, overnight) and washing, the membrane was incubated either with IgY antibodies specific to cADA or control IgYs (10 μg/ml, 1 h, 37 °C). The ADA-IgY complexes were detected by anti-IgY rabbit IgG-HRP antibodies (1 h, 37 °C), and the bands were visualized as previously described, using chemiluminescent substrate.

In order to determine the detection limit of cADA on ELISA with anti-cADA IgY antibodies, a 96-well microtiter plate (MaxiSorp, Nunc, Gdańsk, Poland) was coated with native calf ADA at a concentration range between 1 and 0.005 μg/ml following the protocol described above. Next, the solution of anti-cADA IgYs or control IgY antibodies was added (25 μg/ml in 0.5% skim milk in PBST). After 1 h incubation at 37 °C, the plate was washed with PBST and incubated (1 h, 37 °C) with secondary antibodies conjugated to HRP. The signal development steps were as described above.

### Affinity Purification of cADA-Specific IgY Antibodies

The cyanogen bromide (CNBr)-sepharose resin (Thermo Scientific, Gdańsk, Poland) was packed into the column (Micro Bio-Spin, Bio-Rad, Warsaw, Poland) and extensively washed, first with 1 mM hydrochloric acid, pH 3.0, and next with a coupling buffer (100 mM sodium hydrogen carbonate, 500 mM sodium chloride, pH 8.0) followed by the addition of cADA solution prepared in a coupling buffer (167 μg/ml, 300 μl). After a 2-h incubation at room temperature (with gentle shaking), the reaction was performed at 4 °C (overnight). Next, the resin was washed with Tris-HCl buffer (0.1 M Tris-hydrochloride, 0.5 M sodium chloride, pH 8.3), followed by incubation with Tris-HCl buffer (2 h, room temperature, gentle shaking). The resin was subsequently washed alternately with low-pH buffer (100 mM acetic acid, 100 mM sodium acetate, 500 mM sodium chloride, pH 4) and high-pH buffer (100 mM Tris-hydrochloride, 500 mM sodium chloride, pH 8.0) to elute the unbound cADA. Finally, the resin was washed with PBS and the column was stored at 4 °C until use.

In order to affinity purify anti-cADA IgY antibodies, the crude isolate (150 μl) diluted with PBS (150 μl) was applied to the prepared column containing cADA-modified sepharose and allowed to bind to the target protein (1 h, room temperature). The unbound antibodies were removed by gravity flow, and the resin was washed extensively, first with phosphate buffer supplemented with Tween-20 (10 mM PBS, 25 mM EDTA, 0.1% Tween-20), and next with PBS containing 25 mM EDTA. The anti-cADA IgY antibodies were eluted with 20 mM citrate buffer, pH 2.5. The collected fractions were immediately neutralized with Tris-base buffer (1 M, pH 8.0). After eluting the antibodies, the column was washed with PBS and stored at 4 °C for further use. The presence of IgY antibodies in collected fractions was confirmed via SDS-PAGE under non-reducing conditions, followed by silver staining [55].

Affinity-purified anti-cADA IgYs were further concentrated (Amicon® Ultra Centrifugal Filter; Merck, Warsaw, Poland) and dialyzed against 10 mM PBS. The total protein concentration was determined with Micro BCA assay (Pierce, Gdańsk, Poland), and the cADA-reactivity of purified antibodies was confirmed by the dot-blot analysis. For this purpose, nitrocellulose membrane was coated with cADA (0.5 μg/ml, 100 μl) and blocked with 5% skim milk in PBST (4 °C, overnight). After washing with PBST, the membrane was cut into strips which were incubated with IgY antibodies (1 μg/ml, in 0.5% skim milk in PBST): anti-cADA (crude isolate), affinity-purified anti-cADA, and control. The next steps followed the protocol described above.

### Biotin-Labeled cADA-Specific IgY Antibodies

A 20-fold molar excess of freshly prepared 10 mM solution of biotin (EZ-Link™ NHS-LC-Biotin, Thermo Scientific, Gdańsk, Poland) in dimethylsulfoxide was added to the solution of anti-cADA affinity-purified IgY antibodies in PBS. The reaction was performed for 2 h at room temperature, followed by dialysis against PBS.

### Double IgY Sandwich ELISA for cADA Detection

For a sensitive detection of cADA in a sandwich ELISA format, a 96-well microtiter plate (MaxiSorp, Nunc, Gdańsk, Poland) was coated with anti-cADA affinity-purified IgY or control IgY antibodies in 50 mM sodium carbonate buffer pH 9.6 (2.5 μg/ml, 100 μl/well). After incubation (4 h, 37 °C), the plate was washed with PBST and blocked with 10% skim milk in PBS (4 °C, overnight). Subsequently, the plate was washed and cADA (serial dilution ranging from 0.5 μg/ml to 50 pg/ml in PBS) was added (100 μl/well). After incubation (1 h at 37 °C), the plate was washed and biotinylated anti-cADA IgY antibodies were added (2.5 μg/ml, 0.5% skim milk in PBST). After 1 h incubation at 37 °C, the plate was washed as before and streptavidin HRP (Thermo Scientific, Gdansk, Poland) was added (1:5000 in PBST) followed by 1 h incubation at 37 °C. The plate was developed as described previously. The results were expressed as the OD_490_^*^, where OD_490_^*^ = OD_sample_ − OD_background_.

### Detection of Human ADA Using Anti-cADA IgY Antibodies

Human recombinant ADA_1_ (hADA; diluted 10, 10,0 and 1000 times for silver staining and 50 times for Western blot; Sigma-Aldrich, Poznań, Poland) was resolved on SDS-PAGE, followed by either gel silver staining or electrotransfer onto the nitrocellulose membrane. For Western blot analysis, cADA was used as a positive control (1 ng/well). After blocking and washing, the membrane was incubated with anti-cADA or control IgY antibodies diluted in 0.5% skim milk in PBST (10 μg/ml, 1 h, 37 °C). Subsequently, the membrane was washed in PBST and incubated with secondary antibodies (anti-IgY rabbit IgG-HRP antibodies, 1:5000 dilution in 0.5% skim milk in PBST, 1 h, 37 °C). The signal was developed using chemiluminescent substrate, and the bands were visualized as described before.

For a specific detection of hADA on ELISA, a 96-well microtiter plate was coated with anti-cADA or control IgY antibodies in carbonate buffer pH 9.6 (2.5 μg/ml, overnight, 37 °C), followed by washing with PBST and blocking with 10% skim milk in PBST (4 °C, overnight). After washing, cADA in a dilution range from 0.5 μg/ml to 50 pg/ml and hADA in a dilution range from 100 to 10,000 were added (1 h, 37 °C). After washing with PBST, anti-cADA IgY antibodies conjugated to biotin were added (1.5 μg/ml in 0.5% skim milk in PBST, 1 h, 37 °C), followed by washing with PBST and incubation with streptavidin HRP (1:5000 in PBST, 1 h, 37 °C). The plate was developed as described previously. The results were expressed as the OD_490_^*^, where OD_490_^*^ = OD_sample_ − OD_control_.

### Detection of hADA in Cancer Cell Lysates

All cell lines were purchased from ATTC (Łomianki, Poland) and maintained in the Institute of Immunology and Experimental Therapy Polish Academy of Science (IIET PAS, Wroclaw, Poland). The MOLT-4 cells were cultured in RPMI + HEPES supplemented with 2 mM l-glutamine and 10% fetal bovine serum (all from Sigma-Aldrich, Steinheim, Germany). The HCV-29T and Hu1703He cells were cultured in the mixture of RPMI 1640 and Opti-MEM (1:1, *v*/*v*) medium (Gibco, Paisley, Scotland) supplemented with 2 mM l-glutamine and 5% fetal bovine serum (all from Sigma-Aldrich, Steinheim, Germany). All culture media were additionally supplemented with streptomycin (100 μg/ml; Polfa Tarchomin, Poland) and penicillin (100 U/ml; Polfa Tarchomin, Poland). Cells were cultured at 37 °C under a 5% CO_2_ atmosphere. Culture medium was harvested, and cell culture flasks were soaked with Trypsin-EDTA, pH 8.2 (IIET PAS, Wrocław, Poland), followed by rinsing with PBS and centrifugation (5 min, 4 °C, 300×*g*). Cell pellet was washed three times with PBS. Cells were lysed with RIPA buffer (Sigma-Aldrich, Steinheim, Germany) with the addition of Protease Inhibitor Cocktail (100× dilution, Sigma-Aldrich, Steinheim, Germany) for 15 min at 4 °C. Concentration of total protein in samples was determined using the Micro BCA assay (Pierce, Gdańsk, Poland). Samples were stored at − 22 °C until analysis.

The ability of anti-cADA IgY antibodies to detect ADA expressed in human cell lines was examined by Western blot. For this purpose, calf ADA (from 0.5 to 0.0625 ng/well) and cell lysates (from 40 to 2.5 μg/well of total protein) were resolved by SDS-PAGE (Tris-glycine, 4–12%) and electrotransferred onto a nitrocellulose membrane. For the detection of ADA, cADA-specific IgYs or control antibodies (1 μg/ml in 0.5% skim milk in PBST) were used following the protocol described above.

### Inhibition of cADA with Specific IgY

We performed a microplate-based kinetic assay to verify whether anti-cADA IgY antibodies are able to inhibit the enzymatic activity of cADA [56]. For this purpose, a 96-well microtiter UV-transparent plate (Thermo Scientific, Gdańsk, Poland) was blocked with 2% bovine serum albumin (VWR International, Gdańsk, Poland) in PBS (150 μl/well, 1 h, 37 °C). Next, the plate was washed with PBS (three times, manually, 300 μl/well) and IgY antibodies (specific or control; concentration range from 111.11 to 9.54 nM in PBS buffer, pH 7.2) and cADA (0.04 U/ml) were added to the wells. After 30 min of incubation at 37 °C, adenosine (1.25 mM, PBS buffer, pH 7.2; Carl Roth GmbH, Karlsruhe, Germany) was added. The change of absorbance at 260 nm was monitored on a SpectraMax Plus 384 microplate reader (Molecular Devices, Sunnyvale, CA, USA) for 3 h at room temperature.

## Data Analysis

All data were analyzed using GraphPad Prism version 5.0 software (GraphPad Software Inc., La Jolla, CA, USA). The results are presented as the mean ± SEM of experiments performed in duplicate or as the mean ± SD of two independent experiments performed in duplicate.

## Results and Discussion

### Immunization and Isolation of IgY Antibodies

Hens immunized with cADA emulsified with Freund’s complete (for primary immunization) and incomplete (for booster injections) adjuvant showed no signs of pain or stress over the course of 22 weeks. Antibodies isolated from egg yolks using the PEG 6000 precipitation method showed a purity of 85–90% (as examined by SDS-PAGE followed by a silver staining method) with an average isolation yield of approximately 90 mg per single egg yolk [53].

### Specific IgY Antibody Production

**Fig. 1:**
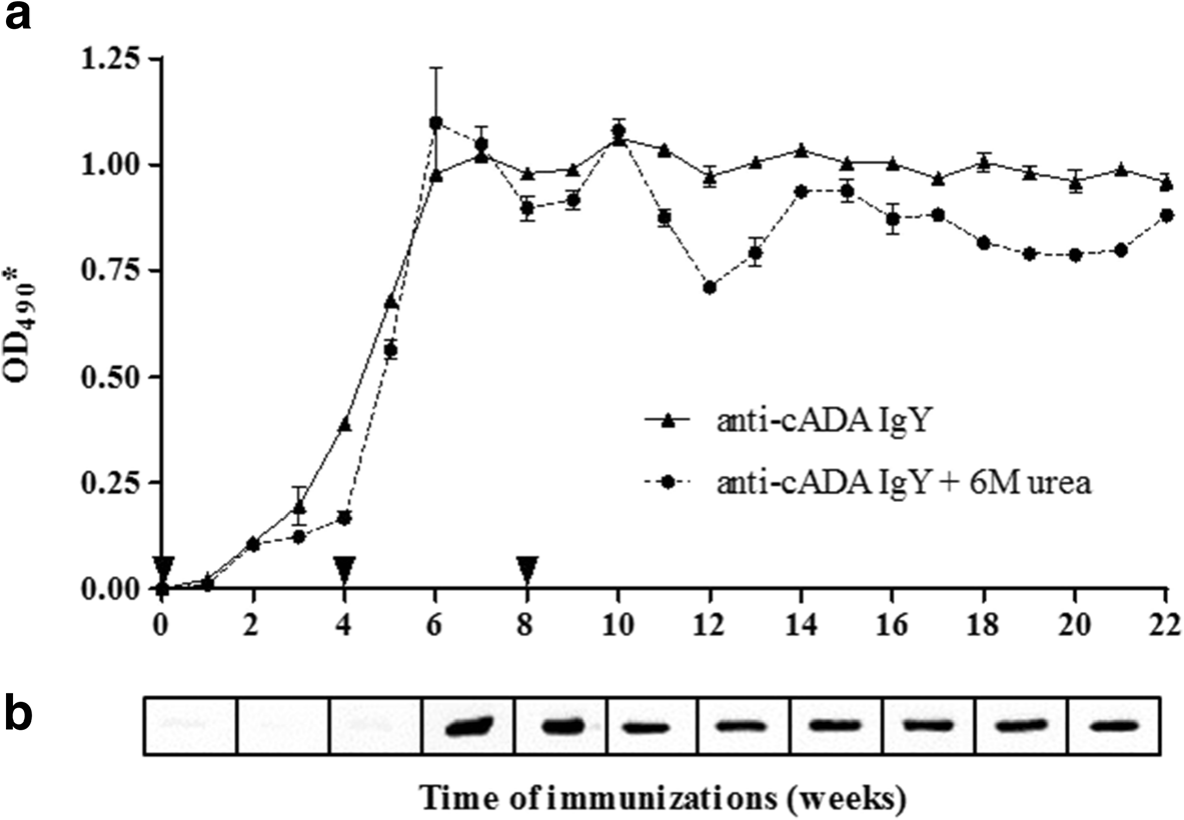
Production of antigen-specific IgY antibodies during the course of immunization. Anti-cADA IgY production (triangles) and avidity (circles) (**a**). For the indirect ELISA, plates were coated with cADA protein (0.5 μg/ml). The time of booster injections is indicated by arrowheads. **b** For Western blot analysis, cADA was used at an amount of 50 ng/lane. In both experiments, anti-cADA antibodies were used at 1:100 dilution and rabbit anti-IgY antibodies were used at 1:5000. The ELISA results are expressed as the OD_490_^*^ ± SEM values obtained after normalization to the background absorbance obtained for the control antibodies

The immune response as manifested by the production of antigen-specific IgY antibodies was monitored during the course of immunization via Western blot and ELISA assays (Fig. 1). Western blot indicated that specific anti-cADA IgY antibodies were present in egg yolks starting from week 6 after the first injection. The ELISA assay showed the presence of antigen-specific IgYs in week 4 while the reactivity of cADA-specific antibodies remained relatively constant from week 6 until the end of the experiment. Similarly, the highest avidity of IgYs was observed from the 6th week and continued to be stable throughout the following weeks.

### Reactivity of cADA-Specific Antibodies

**Fig. 2:**
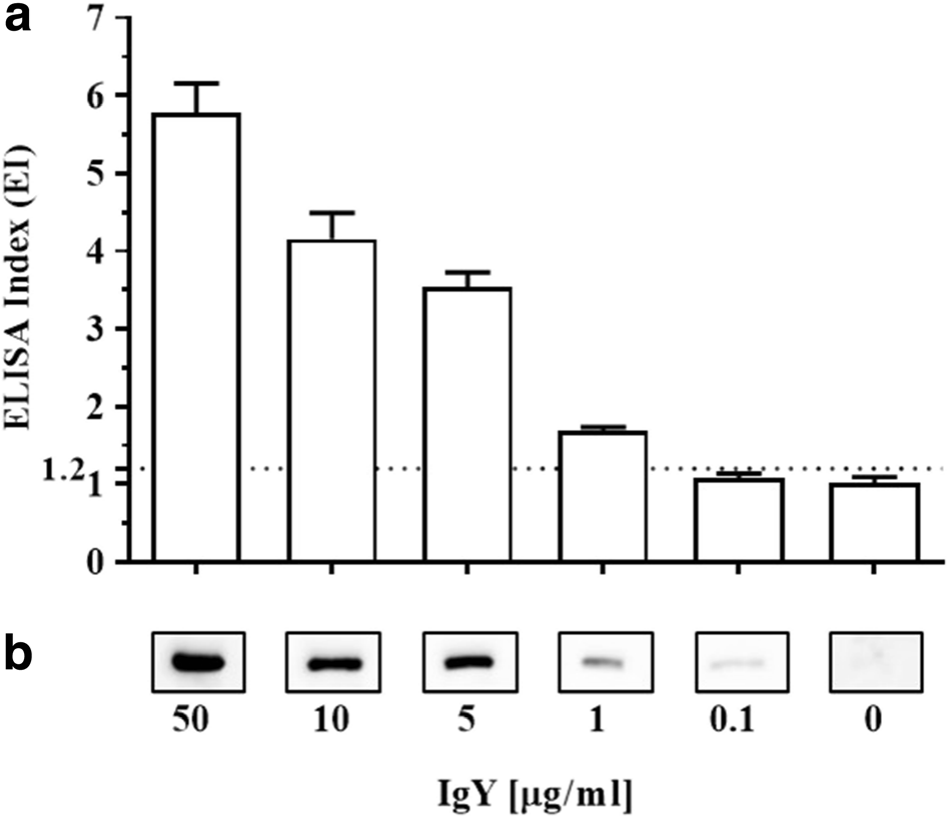
Anti-cADA IgY antibody titer. For indirect ELISA, the plate was coated with cADA antigen (0.5 μg/ml, 100 μl/well) (**a**). For Western blot analysis, 50 ng cADA/lane was used (**b**). In both experiments, IgY antibodies were used in serial dilutions from 50 to 0.1 μg/ml. Rabbit anti-IgY-HRP conjugate was used in a 1:5000 dilution. For Western blot analysis, membrane strips corresponding to 0 were incubated with control IgY antibodies at a concentration of 50 μg/ml. The results of ELISA are presented as an ELISA index (EI) ± SEM, where EI = OD_sample_/OD_control_

**Fig. 3:**
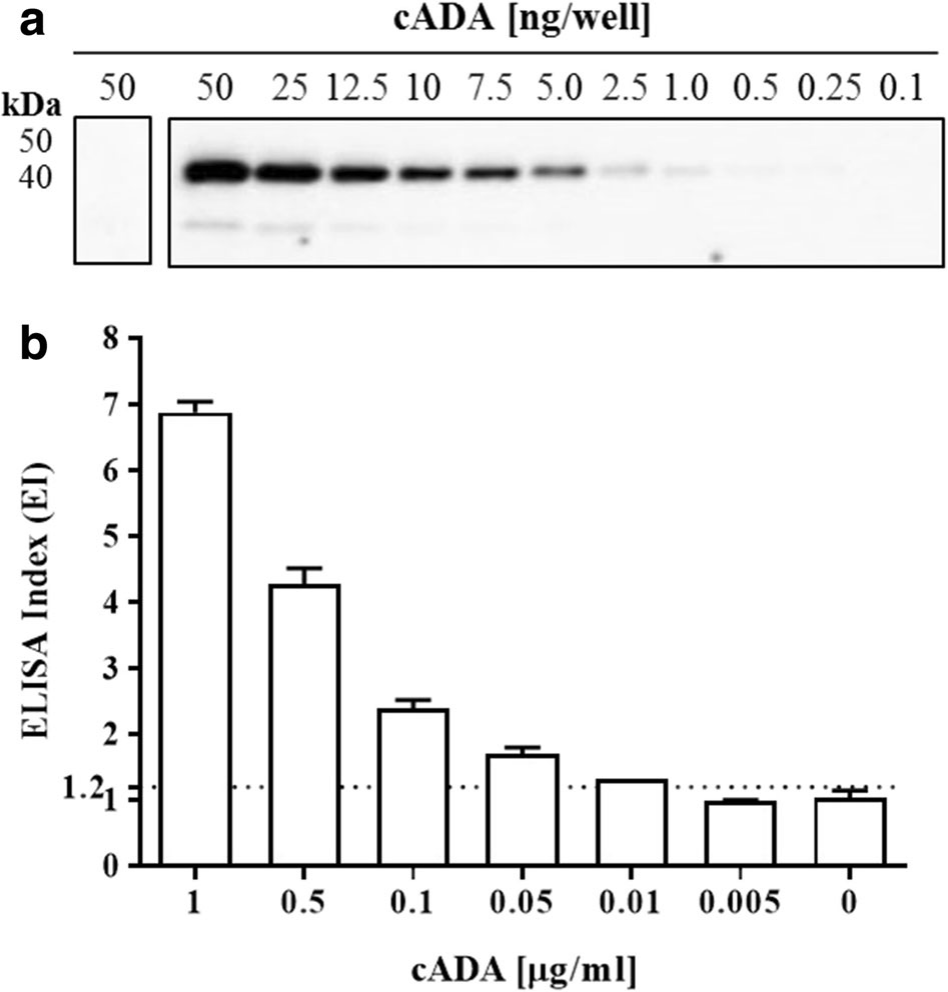
The detection limit of calf adenosine deaminase. For determination of the cADA detection limit, Western blot with target antigen from 50 to 0.1 ng/lane was performed. The membrane was incubated with IgY antibodies (10 μg/ml), followed by rabbit anti-IgY conjugate dilution 1:5000 (**a**). For ELISA, the plate was coated with 1 to 0.005 μg/ml of native calf ADA protein and incubated with antigen-specific and control IgY antibodies (25 μg/ml). Detection of cADA-IgY antibody complexes was performed with rabbit anti-IgY IgG antibodies conjugated with HRP (1:5000) (**b**). For ELISA, the results are presented as an ELISA index (EI) ± SEM, where EI = OD_sample_/OD_control_

The titer analysis of anti-cADA IgY antibodies determined by Western blot showed that the specific band corresponding to cADA (50 ng/well) was detected at an IgY concentration of 0.1 μg/ml (Fig. 2b) whereas for ELISA 50 ng of cADA (0.5 μg/ml, 100 μl) coated on microtiter plate wells was detected with IgYs at 1.0 μg/ml, displaying an ELISA index (EI) greater than 1.2 (Fig. 2a). Western blot analysis revealed the cADA detection limit equal to 1 ng/lane (Fig. 3a). In this experiment, anti-cADA IgY antibodies as well as control antibodies were used at a concentration of 10 μg/ml.

Electrophoretic analysis of ADA purified from calf intestine revealed the presence of two bands (40.3 kDa, 33.3 kDa; Fig. 3a) which is in agreement with previous findings by Cory et al. [57]. Similarly to the result obtained after Western blot analysis, the cADA detection limit on ELISA was equal to 0.1 μg/ml with an EI > 1.2 (Fig. 3b).

### Affinity Purification of Anti-cADA IgY Antibodies

**Fig. 4:**
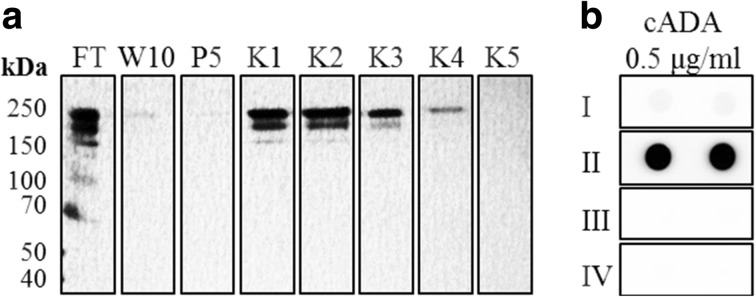
Affinity chromatography purification of anti-cADA IgY antibodies. **a** Standard SDS-PAGE electrophoresis in non-reducing conditions, followed by silver staining. Fractions: FT—flowthrough 25 times diluted in PBS, W10—PBST with EDTA buffer, pH 7.4 (10th wash), P5—PBS with EDTA buffer, pH 7.4 (5th wash), K1–K5 fractions after elution with citrate buffer, pH 2.5. **b** Dot-blot analysis of obtained anti-cADA affinity-purificated antibodies. Strip I—anti-cADA crude IgY antibodies isolate, II—anti-cADA affinity-purified IgY antibodies, III—control IgY antibodies, IV—buffer only. All IgYs were used at a concentration of 1 μg/ml. Protein-antibody complexes were detected with anti-IgY IgG HRP conjugates diluted 1:5000

In order to enrich for specific IgY antibodies that could be potentially used in diagnostic assays after their modification with biotin, an affinity purification step was performed. For this purpose, cADA was conjugated to CNBr-sepharose resin and packed into the chromatography column. After the crude isolates of IgY antibodies were passed through the column, the unbound non-specific IgYs were washed away and cADA-specific IgYs were eluted with low pH buffer. The presence of IgY antibodies in all fractions was confirmed by SDS-PAGE under non-reducing conditions, followed by silver staining (Fig. 4a). Fractions K1–K4 containing anti-cADA IgYs were pooled, concentrated, and dialyzed against PBS. The enrichment with specific antibodies in the sample was confirmed by a dot-blot assay in which a membrane coated with cADA was incubated with non-purified and affinity-purified IgY antibodies (1 μg/ml) as well as with control antibodies (Fig. 4b). The intensity of the signal obtained after the affinity purification was approximately 7.6 times higher than the intensity of the signal obtained for unpurified antibodies. We have also noticed that supplementing the phosphate buffer with EDTA (25 mM) significantly increased the lifespan of the resin which could be re-used at least 40 times.

### Development of a Sandwich ELISA for a Specific Detection of cADA

**Fig. 5:**
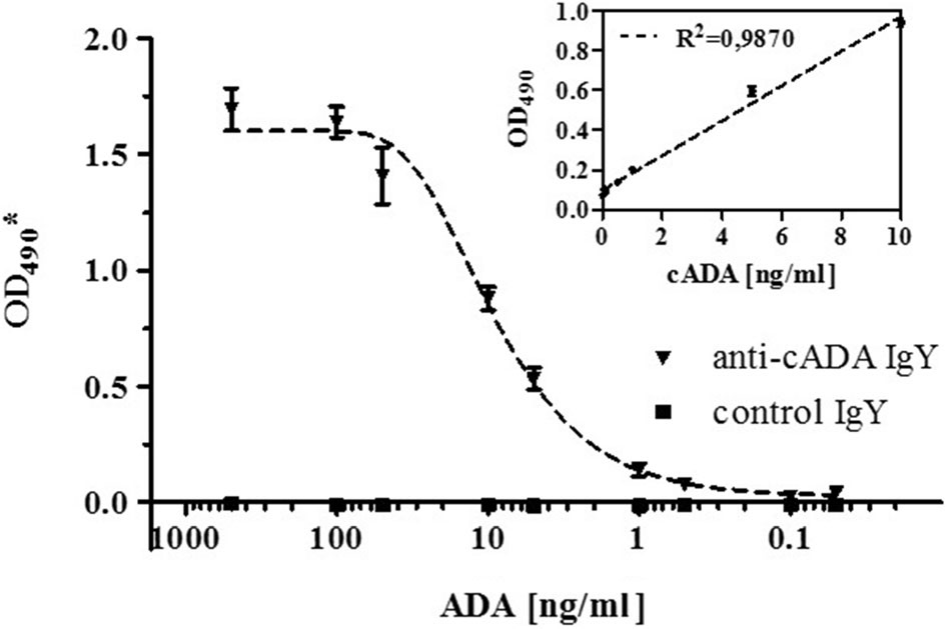
Sandwich-type ELISA assay for calf adenosine deaminase detection. The plate was coated with anti-cADA affinity-purified IgY antibodies and control IgY antibodies (2.5 μg/ml) followed by incubation with cADA at concentrations ranging from 500 to 0.05 ng/ml. For detection, affinity-purified anti-cADA IgY biotin-labeled antibodies were used at a concentration of 2.5 μg/ml. The complexes were detected with streptavidin conjugate with HRP (1: 5000 dilution). Symbols represent mean ± SD from two independent experiments performed in duplicate for each point and are expressed as the OD_490_^*^ values obtained after subtraction of the background values

For a sensitive and specific detection of cADA, a sandwich-type ELISA has been developed with the use of polyclonal affinity-purified anti-cADA IgY antibodies as coating antibodies and their biotin-labeled form as detection antibodies (both at concentration of 2.5 μg/ml). Using this system, cADA was successfully detected at a concentration of 0.5 ng/ml with the linearity range up to 10 ng/ml (Fig. 5). Such a sandwich-type assay for a sensitive detection of ADA is in a format preferable for serological diagnostics and could provide an interesting alternative to the commonly used ADA tests which rely on activity measurements.

### Detection of Human ADA

**Fig. 6:**
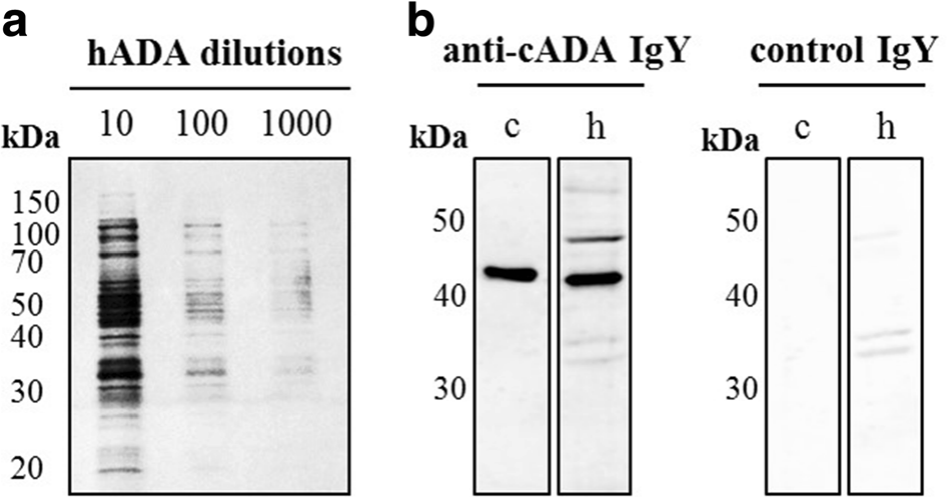
The detection of human recombinant adenosine deaminase by anti-calf adenosine deaminase IgY antibodies. Human ADA (10, 100, and 1000 times diluted; reducing conditions) was resolved on SDS-PAGE, followed by silver staining. SDS-PAGE (reducing conditions, 4–12% Tris-glycine) of calf (1 ng/well) and human (50 times diluted) ADA followed by nitrocellulose electrotransfer was performed. The membrane was incubated with anti-cADA and control IgY antibodies (10 μg/ml). Protein-antibody complexes were detected with anti-IgY IgG HRP conjugates diluted 1:5000

**Fig. 7:**
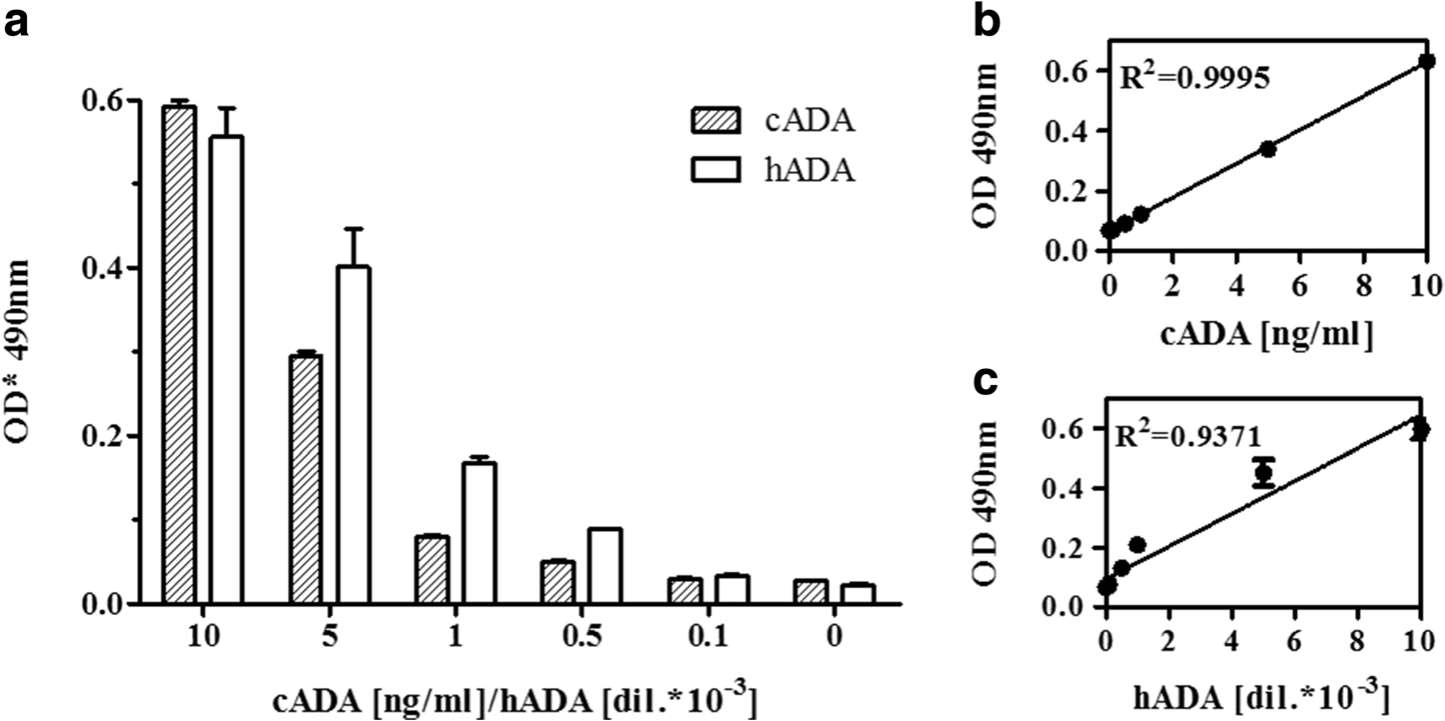
Double IgY sandwich ELISA for sensitive detection of adenosine deaminase. Anti-cADA and control IgY were used as coating antibodies (2.5 μg/ml in carbonate buffer). cADA in the concentration range from 10 to 0.1 ng/ml and hADA in a dilution range from 10 to 10,000 were used, followed by incubation with anti-cADA IgY antibodies modified with biotin at a concentration of 2.5 μg/ml. Complexes were detected with the use of sterptavidin HRP conjugate (1:5000). Results are expressed as the OD_490_^*^ ± SEM values obtained after subtraction of the values recorded for the control antibodies

IgY antibodies specific to cADA were used for specific detection of recombinant human ADA in a highly complex commercial sample (enzymatically active, human ADA_1_, recombinant, expressed in *E. coli*, Figs. 6 and 7). Although there is a high homology between mammalian ADAs, a comparison of hADA and cADA amino acid sequences revealed some differences located mainly within the α-helices present on the surface of both proteins. Such an exposition of structurally different epitopes might explain the observed differences in the recognition of cADA and hADA by generated IgY antibodies in a sandwich-type ELISA (Fig. 7). Additional bands observed on Western blot analysis in a highly complex human ADA sample (Fig. 6) may suggest that anti-cADA IgYs are not 100% specific.

### Detection of hADA in Lysate of Different Human Cancer Cell Lines

**Fig. 8:**
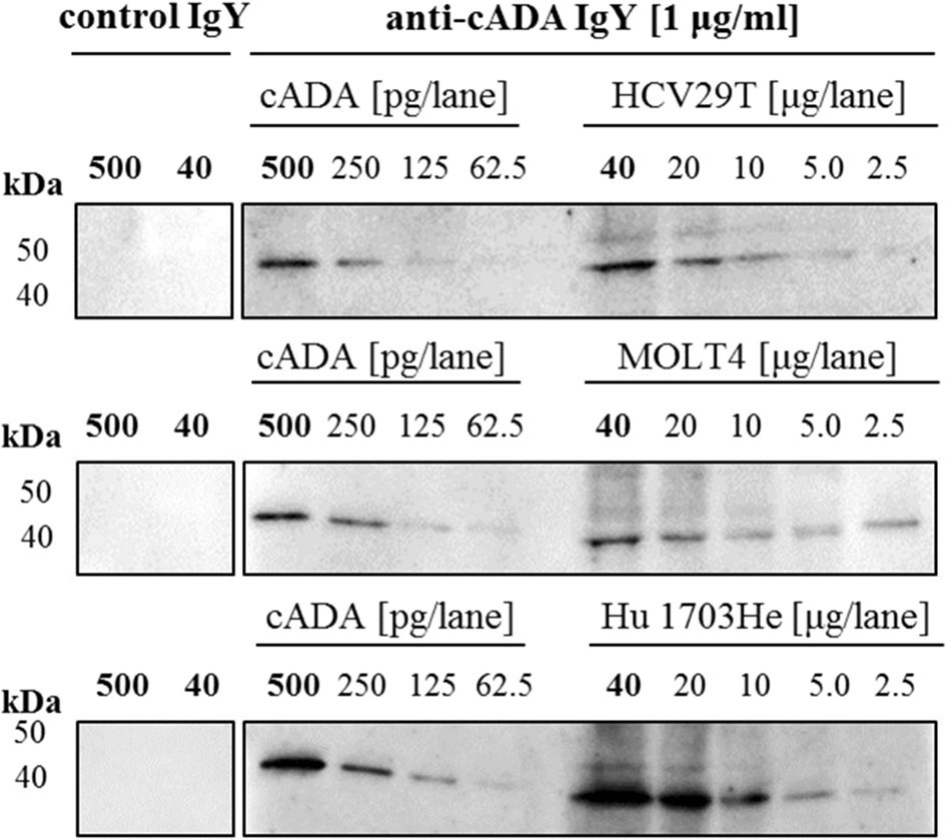
The detection of adenosine deaminase in human cell lines. For determination of the ability of IgY antibodies to detect ADA in human cell line lysates, cADA at a concentration range from 500 to 62.5 ng/lane (as a positive control) and cell lysates (total protein concentration from 40 to 2.5 μg/lane) were resolved on SDS-PAGE (4–12%, Tris-glycine). After electrotransfer, the membrane was incubated with specific and control IgY antibodies (1 μg/ml), followed by an incubation with rabbit anti-IgY conjugate diluted 1:5000

The detection of hADA in cell lysates on Western blots indicated that IgY antibodies are able to detect picograms of target protein in complex cell lysate samples with a total protein concentration ranging between 10 and 40 μg/lane. Based on the intensity of cADA bands, the amount of hADA in 10 μg of MOLT4, Hu 1703He, and HCV92T cell lysate ranges between 125 and 500 pg (Fig. 8).

The species cross-reactivity of anti-cADA IgY antibodies allows for future development of sensitive assays aimed at detecting hADA.

### Inhibition of Calf ADA Activity by Specific IgY Antibodies

**Fig. 9:**
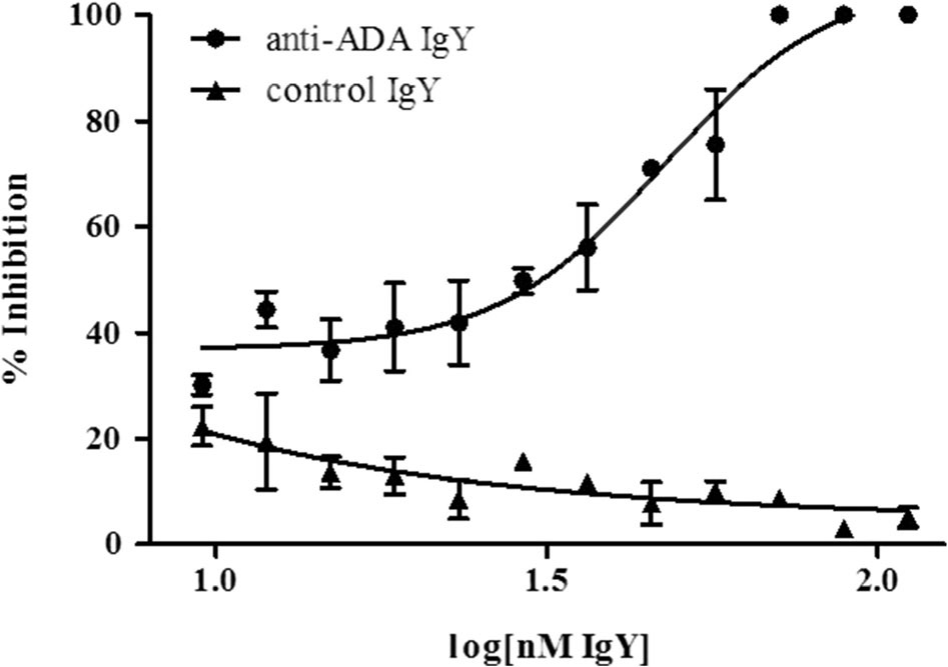
Evaluation of anti-calf adenosine deaminase affinity-purified IgY antibodies as an inhibitor of adenosine deaminase. IgY antibodies (specific and control) at final concentrations ranging from 111.11 to 9.54 nM were mixed with native cADA (final concentration of 0.04 U/ml) in PBS buffer, pH 7.2. After 1 h incubation at 37 °C, adenosine in PBS was added (final concentration 1.25 mM) and the deamination reaction was monitored (A_260_). For calculation of the IC_50_, a value variable slope equation model was applied. Symbols represent mean ± SD from two independent experiments performed in duplicate for each point

The influence of affinity-purified antigen-specific and control IgY antibodies on the enzymatic activity of cADA was examined with a microtiter-based kinetic assay [56]. IgY antibodies at concentrations ranging from 111.11 to 9.54 nM were incubated with native cADA (0.04 U/ml) for 30 min, followed by the addition of substrate, and the change in absorbance was monitored for 3 h. To calculate the IC_50_ value, a four-parameter dose-response curve model (equation: log(inhibitor) vs. response-variable slope model) was applied and a sigmoid curve model was fit to the data (*R* ^2^ = 0.9485) [58, 59]. The determined IC_50_ value for specific anti-cADA IgY antibodies was 47.58 nM, while for the control IgYs no inhibitory effect on cADA activity was observed (Fig. 9). It is hard to speculate whether the application of IgY inhibitors as potential therapeutic agents is feasible; however, the design and production of IgY antibodies that affect the growth of *H. pylori* as enzyme inhibitors appear to be promising [60–62].

## Conclusions

The potential application of ADA as a diagnostic marker of various types of cancer including breast, bladder, ovary, tongue, and intestine highlights the usefulness of the method presented here [22–24, 28, 29]. The developed IgY-based sensitive ADA detection assay applies polyclonal hen egg-yolk antibodies for capture and detection of the target antigen in a sandwich ELISA format. The anti-cADA IgY antibodies were able to specifically recognize ADA in human cancer cell lysates. Such cross-reactivity of IgY antibodies obtained through immunization of hens with calf ADA is only possible due to a high homology between both proteins. Considering the fact that current ADA diagnostic testing relies mainly on the measurement of its enzymatic activity, based on the Giusti and Galanti method, our proposed assay could provide an alternative diagnostic option [63]. The studies regarding this are ongoing in our laboratory.

## Abbreviations

ADA: Adenosine deaminase
ADA_1_-S: Adenosine deaminase small form
ADA_1_-L: Adenosine deaminase large form
cADA: Calf adenosine deaminase
hADA: Human adenosine deaminase
BLAST: Basic Local Alignment Search Tool
CA 15-3: Cancer antigen 15-3
DPPIV: Dipeptidyl-dipeptidase
EDTA: Ethylenediaminetetraacetic acid
EHNA: Erythro-9-(2-hydroxy-3-nonyl)adenine
EI: ELISA index
ELISA: Enzyme-linked immunosorbent assay
HAMA: Human anti-mouse antibody
HER2: Human epidermal growth factor family receptor-2/Neu
HRP: Horseradish peroxidase
KLK3: Kallikrein 3
KLK6: Kallikrein 6
NHS-LC-Biotin: Succinimidyl-6-(biotinamido) hexanoate
PBS: Phosphate-buffered saline
PBST: Phosphate-buffered saline with Tween
PDB: Protein database
PEG: Poly(ethylene glycol)
RNR: Ribonucleotide reductase
SDS-PAGE: Polyacrylamide gel electrophoresis with sodium dodecyl sulfate
